# Association Between Dental Expenditure and Socioeconomic Status in Iran

**DOI:** 10.1016/j.identj.2024.04.027

**Published:** 2024-06-08

**Authors:** Mohammad-Pooyan Jadidfard, A. Hamid Zafarmand, Sediqe Shafiei

**Affiliations:** Department of Community Oral Health, School of Dentistry, Shahid Beheshti University of Medical Sciences, Tehran, Iran

**Keywords:** Dental care, Health expenditure, Socioeconomic factors, Gamma regression

## Abstract

**Background:**

Health care spending by households can be a great indicator of a society's commitment to good health stewardship and the efficiency of institutions responsible for managing health costs. Equitable and appropriate distribution of dental services is a challenging issue for realising universal health coverage. This study aimed to evaluate Iranian households’ per capita dental expenditure (DE) according to their socioeconomic status (SES).

**Methods:**

In this cross-sectional study, the income and expenditure of 18,701 urban and 19,261 rural households in Iran were scrutinised according to the data provided by the Statistics Center of Iran (2017-2018). After model creation, the SES index was determined using principal component analysis and weighting based on the analytical hierarchy process. The dependent variable was the share of per capita household's expenditure spent on dental health. The zero-inflated gamma regression model was applied to confirm the presumed association between per capita DE and SES. Analyses were performed using PROC NLMIXED in SAS software (version 4.9).

**Results:**

The results revealed that approximately 9% of urban and 4% of rural households paid for dental treatments in the past month. The DE to total health expenditure (HE) ratios were 8.5% and 14.8% for rural and urban households, respectively. Also, with each level increase in SES, the average per capita DE increased by 23% and 16% in rural and urban households, respectively.

**Conclusions:**

The study confirms association between per capita DE and SES in Iran. This implies targeted strategies to facilitate the utilisation of dental care especially for lower SES groups according to their needs.

## Background

Dental expenses amount to a total of over $300 billion per year globally, accounting for 4.6% of all health care expenditures (HE), making oral and dental services the fourth most costly medical care.^1^^,^^2^ In high-income countries, approximately 5% to 10% of the health budget is spent on dental care^3^; however, not much budget is allocated to dental care in lower- and middle-income countries, where this tight budget is mainly spent on emergency care, and the costs of health care services are mainly paid out of pocket.^1^^,^^4^^,^^5^

Iran is a country in West Asia with an estimated population of around 87 million as of 2023 (the world's 17th most populous country). Iran is the second largest country in the Middle East, and according to the World Bank, the country is placed under the low-middle income stratum.^6^ In Iran, 2 types of dental care services are available: public and private. The public oral health care sector in Iran is called the ‘Health and Treatment Network’ and provides oral examination/screening services, oral health education, fluoride varnish application, fissure sealants, tooth scaling and polishing, tooth extraction, restorative treatments, and pulpotomy.^7^ The village insurance in Iran covers all the above-mentioned oral health services for free, while residents in cities must pay 30% of the fee. It should be noted that more than 74% of Iranians reside in cities. The public sector in the country covers approximately 20% and 70% of dental services in metropolitan regions and rural areas, respectively.^8^ In 2015, Iran's government extensively revised the health care system by introducing the Health System Reform Plan, aiming to improve the insurance coverage of health care services to balance out-of-pocket payments.^8^ Despite the incorporation of oral health in basic health care services and the implementation of an oral health reform project, out-of-pocket payments for dental care remain high in Iran, indicating the inadequacy and insufficiency of the aforementioned strategies in reducing the financial burden of oral health services for Iranian households.^9^

Evidence shows that financing dental services affects their utilisation,^10^ and the costs and affordability of dental services are prominent barriers to their accessibility.^11^ Thus, unaffordable out-of-pocket payments will certainly deprive people of receiving the optimal oral health services that they may need. Otherwise, a high percentage of the family's income is devoured by such services, which brings about adverse financial consequences for the family.^12^ In light of the above-mentioned issues, alternative mechanisms and strategic planning need to be developed to financially empower low-socioeconomic status (SES) families,^13^ which demands accurate information about financial indicators, such as household spending on dental care. In fact, the lack of adequate information impedes health authorities from implementing efficient policies.

A recent study^14^ reported that, on average, 8% of Iranian households had payments for dental care during the month before recruitment; however, this study did not consider independent analyses on urban and rural households, reported only a limited number of SES indices, and assumed income and education as proxies for SES, a practice that has been declared unsuitable for developing countries like Iran.^15^ When it comes to health care costs, catastrophic health care expenditures have extensively been discussed, yet exact household spending on health care services has not been adequately addressed.^16^ Thus, this study was conducted to investigate the association between SES and per capita dental expenditure (DE) in Iranian rural and urban households using an adapted SES indicator.

## Methods

### Study design and data collection

This was a cross-sectional study with secondary-based data analysis. Households’ income and expenditures data were collected by the Statistical Center of Iran. The data collection period was from 21 April 2017, to 20 April 2018. In this research, all samples of the statistical centre were involved in the study, and the research population included 18,701 urban households (64,144 people) and 19,261 rural households (69,725 people). The households were selected using probability sampling methods. A 3-stage cluster sampling method was used. First, out of 31 provinces, 2 strata (urban and rural) areas were defined. In each strata, cluster sampling was performed. About 3294 blocks from urban and 3350 from rural areas were specified by random sampling and based on the probability proportional to the size of population. Finally from each block six household were selected by systematic sampling. The questionnaire was completed by face-to-face interviews with household members. Based on the data provided by the Statistics Center of Iran, the researcher defined costs and employed the responses related to DE and SES.

This study's protocols were approved by the Ethics Committees for Research of Shahid Beheshti University of Medical Sciences (ethics code: IR.SBMU.RIDS.REC.1396.447).

### Data collection questionnaire

We used the questionnaire of Classification of Individual Consumption by Purpose (COICOP). This tool has been translated and adjusted by the experts of the Statistics Center of Iran and is standardised every year according to the latest changes in Iranian communities. This questionnaire includes four domains: (1) social features of family members, (2) home property status and its facilities, (3) expenditure on food and non-food items, and (4) the household's income. The third domain of the questionnaire (expenditure on food and non-food items) comprised 14 sections, including information on HE in the recent month. DE is defined as a subset of comprehensive HE that reflects the costs paid for any oral health care services (visits to dentists, tooth extraction, tooth restoration, scaling and root planning, prosthodontics, orthodontics, root canal therapy, implantation, and dental/periodontal surgery) received in governmental or non-governmental centres in the mentioned period. It should be noted that, the household HE and DE converted to per capita expenditure by dividing by the number of person in the household.

### The SES index

We here used an adopted SES index developed in our previous studies.^17^^,^^18^ This index was developed after modelling and implementing principal component analysis and weighting using the analytical hierarchy process. The SES of rural households was determined according to the education level and occupation of the main breadwinner, as well as the family's size, household income, the number of rooms in the house, the surface area of the household's property, possession of a personal computer, ownership of a car, and on fruits and meat. All of the above-mentioned items are considered as a potential property for urban households, except for the family's size, with the addition of the ownership of a dishwasher and microwave.^17^^,^^18^

The SES index for each household ranged from 0 to 1, and it was divided into 10 categories with 0.1 intervals. Subsequently, the SES was re-scaled to a 0 to 100 scale for the regression analysis in order to be adopted to per capita DEs. This adjustment ensured that each level increase in the analysis corresponded to a 0.1 increase in the original scale. According to ordered tables with regression coefficients, the values of Exp. (beta) were calculated using antilogarithm functions based on the Napier number (exp). Interpretations were made based on decimals in both zero and gamma regression models after raising Exp. (beta) values to the power of 10.

### Statistical analysis

Two approaches were used for statistical analysis:

A. Based on the nature of the response variable (per capita DE), which is a continuous quantitative variable and positive, the gamma regression model was used to analyse the data. Given the nature of our data (continuous non-decreasing variables), conventional models, such as linear least squares regression analysis, frequently result in the poor estimation of explanatory variable coefficients. Thus, in order to more reliably estimate the household's expenditure, alternative models, such as the gamma regression model, are required.^19^
B. Studies on HE data often show a limited range of deviation and frequent zero values (i.e., zero mass). This causes the data to have a distribution skewed to the right.^20^ Regarding the issue of zero cost, the 2-part model is probably the most informative and applicable. Therefore, the zero-inflated gamma regression model was employed to assess the correlation between per capita DE and SES. In zero-inflated models, 2 types of zeros are considered in the data: (1) sampling zeros that are related to the time of sampling, representing individuals who did not seek any dental treatment during the study period, and (2) structural zeros, representing those who have never sought any dental treatment.^21^

In the first phase of the study, the possibility of per capita DE being structurally zero was modelled using a logit link function, the coefficients of which were interpreted similarly to those of logistic regression. In the second phase, per capita DE (which was considered a positive value) was modelled using gamma regression and the log link function, the coefficients of which were interpreted similarly to those of Poisson regression). Analyses were conducted using Proc NLMIXED (SAS software version 4.9).

## Results

The data showed that in 2017, approximately 9% of urban households and 4% of rural households in Iran sought oral health services in the month prior to the study. Further analysis was conducted to determine per capita DE, HE, and the share of DE from HE separately in urban and rural households. Accordingly, the share of DE from HE was 14.8% in urban households and 8.5% in rural households (Table 1).

**Table 1:** Per capita expenditure in Iranian rural and urban households.

Variables	Mean Urban households	Mean Rural households
*HE per capita	6,900,000 ^R^ 172.5 ^$^	4,163,000 ^R^ 104 ^$^
*DE per capita	1,020,000 ^R^ 25.5 ^$^	352,000 ^R^ 8.8 ^$^
Share of DE from HE	14.8%	8.5%

⁎ The expenditures are calculated for 1 year. R: Rial; $: US Dollar exchange.

As shown in Appendix 1, the SES index was obtained as 0.30 ± 0.188 in urban households, which was higher than that of rural households (0.18 ± 0.096). The median SES was 0.29 for the urban population and 0.17 for the rural population.

**a. Rural households**

According to Table 2, in the zero part of the model, SES promotion from one level to a higher one, the odds of per capita DE not being zero increased by approximately 57% [Exp. (10*beta) = 0.956^10^ = 0.638, 1/0.638 = 1.567] (*P*-value < .001). Regarding gamma regression coefficients (i.e., positive continuous values), for each level increase in SES, per capita DE increased by 23% on average [Exp. (10*beta) = 1.021^10^ = 1.231] (*P*-value < .001).

**Table 2:** Regression coefficients of the zero-inflated gamma model based on SES and per capita dental expenditures in rural households.

Model	Parameter	Estimate	Exp (Beta)	Standard error	Pr > |t|
**Zero part** (Logistic)	Intercept	4.4158	82.75	0.09588	<.0001
	SES	−0.04496	0.956*	0.003861	<.0001
**Non**-**zero part** (Gamma)	Intercept	1.6971	5.458	0.1233	<.0001
	SES	0.02052	1.021†	0.004700	<.0001

⁎ Odds ratio.

† Rate ratio.

**b. Urban households**

In the zero part of the model, SES promotion from one level to a higher one, the odds of per capita DE not being zero increased by almost 30% [Exp (10*beta) = 0.974^10^ = 0.768, 1/0.768 = 1.302] (*P*-value < .001, Table 3). Regarding the gamma part of the model (i.e., positive continuous values), each level increase in SES predicted an average increase of 16% in per capita DE [Exp (10*beta) = 1.015^10^ = 1.160] (*P*-value < .001).

**Table 3:** Regression coefficients of the zero-inflated gamma model based on SES and per capita dental expenditures in urban households.

Model	Parameter	Estimate	Exp (Beta)	Standard error	Pr > |t|
**Zero part** (Logistic)	Intercept	3.7722	43.475	0.07063	<.0001
	SES	−0.02642	0.974*	0.001645	<.0001
**Non**-**zero part** (Gamma)	Intercept	2.1029	8.189	0.08655	<.0001
	SES	0.01530	1.015†	0.001974	<.0001

⁎ Odds ratio.

† Rate ratio.

## Discussion

The aim of this study was to determine the share of DE from the HE of Iranian rural and urban households and examine the correlation between per capita DE and the household's SES. According to the findings of a 2017-2018 survey, about 9% of urban households and 4% of rural households in Iran incurred costs for dental care, which was in line with the results of other studies conducted in Iran.^14^^,^^22^ Another study in France showed a similar correlation between DE and SES, which was consistent with our findings in a national survey in Iran.^11^

This study demonstrated the difference between the DE to total HE ratios, in rural and urban households (8.5% and 14.8%). This difference may be owing to several factors such as lower access of rural families to dental services and lower levels of SES in rural households compared to urban households.

An increase in accessibility to health care networks and trained dentists has caused dental services to be available in most urban and rural areas of Iran.^8^ However, the affordability of these services is a significant barrier to oral health promotion despite acceptable accessibility. This indicates that most households have limited financial resources to spend on oral health care. Yet, it should not be neglected that the level of health literacy is another factor affecting seeking oral health care. If families cannot afford these services, they will be either denied from receiving a particular service or forced to choose a less expensive treatment option (such as tooth extraction). According to studies conducted in Iran, dental care costs are categorised under catastrophic health expenditures.^23^, ^24^, ^25^ Also, a study on 41 low- and middle-income countries demonstrated that urban households with higher SES spent higher DE and catastrophic health expenditures.^26^ Nevertheless, since most oral and dental complications are not often life-threatening, it appears that the concept of catastrophic expenditures cannot be attributed to routine dental treatments.^27^ Actually, seeking less expensive oral health care for any of the above-mentioned reasons was confirmed by the results of a national survey on dental caries in Iran with respect to the **D**) decayed teeth) and **M** (missed teeth) components of DMFT, with the share of the **M** and **D** components being increased with age.^8^ Therefore, the ratio of zeros regarding DE in Iranian households (i.e., refusal of treatment) observed in the present study could be partially explained by this notion, as well as the variable definitions pertaining to the last month's status.

According to our results, each level increase in the SES boosted the means of per capita DE spent by Iranian rural and urban households, affirming the results of other studies conducted in Iran.^14^^,^^22^^,^^28^ The direct association between the household's SES and HE can be due to the higher affordability and utilisation of services.^29^ People with lower SES receive fewer health care services despite the fact that they often have more needs for such services (the ‘inverse care law’).^15^^,^^22^^,^^30^^,^^31^ It has been demonstrated that in lower- and middle-income countries, health care costs are mostly paid out-of-pocket, so the utilisation of such services is largely governed by the financial power of households rather than their actual needs.^26^

We found a significant difference in the mean per capita DE between rural and urban households. In other words, each level increase in the SES increased the mean per capita DE by 23% in rural households and 16% in urban households. This difference can be partly explained by the ‘cost-outcome curve’ introduced by Robert G. Evans.^32^ Overall, our study showed a lower SES in rural households compared with urban households (Appendix 1). The relationship between the per capita DE and SES in rural households resembled the steep part of the Robert G. Evans curve, meaning that a significant difference in the mean expenditure could be expected from one level to another (either upward or downward). The pattern of the SES-DE relationship in urban households was similar to the flatter part of the above-mentioned curve, indicating a milder change in the mean expenditure with transitioning between SES levels in urban households (Figure).

**Figure:**
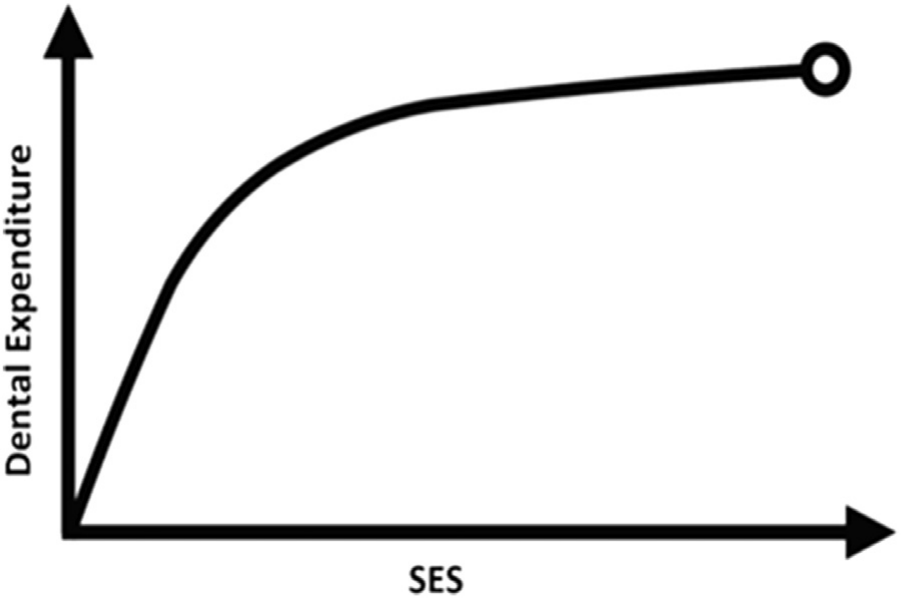
The cost-outcome curve representing dental expenditure and the SES index.

Out-of-pocket payments for dental care can impose a significant financial burden on families, resulting in treatment refusal. Therefore, it is recommended to expand the insurance coverage and governmental support to reduce catastrophic health expenditures and subsequently promote oral health. More importantly, targeted strategies for financially supporting patients with lower SES are also recommended to eliminate the financial barriers to the effective provision of essential oral health services.

### Limitations

Our analyses were based on the data gathered by the Statistics Center of Iran and focused on the household's HEs in 1 month preceding the study. So, there were many zero values because of the limited period of the national survey. It should be noted that this is a common drawback in all national surveys conducted in many countries. Also, there were some worth-mentioning covariates, including oral health behaviours and oral health status that are recommended to be included in national survey checklists to clarify the association between SES and per capita DE. The lack of incorporation of such covariates as well as the possibility of measurement errors in SES may potentially lead to bias in research findings.

## Conclusion

The study confirms the theoretically approved association between per capita DE and SES in the low-middle income context of Iran. This implies targeted strategies to facilitate the utilisation of dental care especially for lower SES groups (who bear more burden of disease) according to their needs. Future research can clarify more details of such association particularly by explaining the role of mediate factors such as behavioural ones, to inform more effective policies at different levels of decision-making.

## Appendix Supplementary materials

Image, application 1

Image, application 2
