# Supplementary material for: Association Between Dental Expenditure and Socioeconomic Status in Iran
Source: Int Dent J. 2024 Jun 8;74(6):1432–7. doi: 10.1016/j.identj.2024.04.027 (PMC11551582; doi:10.1016/j.identj.2024.04.027)
Supplement: Supplementary file 1 [file mmc1.docx]

**Appendix 1. Descriptive characteristics of Socioeconomic Status Index in urban and rural households**

| **Value** | **Urban** | **Rural** |
| --- | --- | --- |
| N Valid | 18701 | 19261 |
| N Missing | 450 | 7 |
| Mean | .30 | .18 |
| Median | .29 | .17 |
| Std. Deviation | .19 | .096 |
| Mode | .09 | .15 |
| Minimum | .000 | .000 |
| Maximum | .92 | .70 |
| Percentiles 25 | .17 | .11 |
| Percentiles 50 | .29 | .17 |
| Percentiles 75 | .45 | .24 |
